# Reconstructing Seasonal Range Expansion of the Tropical Butterfly, *Heliconius charithonia*, into Texas Using Historical Records

**DOI:** 10.1673/031.010.6901

**Published:** 2010-06-22

**Authors:** Márcio Zikán Cardoso

**Affiliations:** Departamento de Botânica, Ecologia e Zoologia, Centro de Biociências, Universidade Federal do Rio Grande do Norte, Brazil

**Keywords:** abiotic factors, dispersal, geographical range, Nymphalidae, range limits

## Abstract

While butterfly responses to climate change are well studied, detailed analyses of the seasonal dynamics of range expansion are few. Therefore, the seasonal range expansion of the butterfly *Heliconius charithonia* L. (Lepidoptera: Nymphalidae) was analyzed using a database of sightings and collection records dating from 1884 to 1992 from Texas. First and last sightings for each year were noted, and residency time calculated, for each collection locality. To test whether sighting dates were a consequence of distance from source (defined as the southernmost location of permanent residence), the distance between source and other locations was calculated. Additionally, consistent directional change over time of arrival dates was tested in a well-sampled area (San Antonio). Also, correlations between temperature, rainfall, and butterfly distribution were tested to determine whether butterfly sightings were influenced by climate. Both arrival date and residency interval were influenced by distance from source: butterflies arrived later and residency time was shorter at more distant locations. Butterfly occurrence was correlated with temperature but not rainfall. Residency time was also correlated with temperature but not rainfall. Since temperature follows a north-south gradient this may explain the inverse relationship between residency and distance from entry point. No long-term directional change in arrival dates was found in San Antonio. The biological meaning of these findings is discussed suggesting that naturalist notes can be a useful tool in reconstructing spatial dynamics.

## Introduction

Every year tropical butterflies arrive in temperate North America in a seasonal range expansion driven primarily by seasonal shifts in climate and seasonally-mediated changes in resource availability. Predictable seasonal changes in local climate drive seasonal shifts in the distribution of many tropical butterfly species that arrive in temperate North America from Mexico (maps in Opler and Malikul 1992). In close proximity to tropical and subtropical butterfly ranges, Texas receives many species that migrate from Mexico (Gilbert 1969).

In addition, range shifts due to global climate change are particularly well documented in Europe and in some areas of the United States (Parmesan et al. 1999, Roy and Sparks 2000, Forister and Shapiro 2003, Parmesan 2006). However, the dynamics of these seasonal shifts (regardless of climate change) have yet to be examined in detail. Due to the increasing interest in shifts in range limits and their dynamics, the factors that may influence seasonal movement of a well known tropical butterfly, *Heliconius charithonia* L. in Texas, USA are described and examined using historical data to reconstruct arrival and residency patterns. Additionally information was used from a well-sampled locality to test whether arrival dates have shifted over time, perhaps due to climate change.

Historical reconstruction offers a plausible approach for both discovering ecological patterns and for generating testable hypotheses about those patterns (Sagarin 2002), but may suffer from a variety of limitations (Parmesan et al. 2005). Short times series, difficulties in obtaining adequate spatial replication, and obstacles in interpretation of non significant results will often, but not always, be problems. The reconstruction of the seasonal migrations of *H. charithonia* is a good example of both limitations and uses of such an approach.

## Materials and Methods

### *Heliconius charithonia* natural history

**Figure 1.**
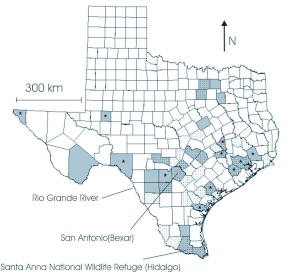
Map of Texas with distribution of *Heliconius charithonia* (gray) and *Dryas iulia* (dotted). Sites with a star are additional counties obtained from BAMA website not present in RK's original data. Sites without a star (*H. charithonia* only) are from both BAMA and RK. Distribution data for *Dryas iulia* is entirely derived from BAMA (www.butterfliesandmoths.org). High quality figures are available online.

*Heliconius charithonia* is a tropical butterfly (family Nymphalidae) found in open and second growth habitats in tropical North and South America and in the Caribbean (Sheppard et al. 1985). In the United States, the subspecies *H. c. vazquezae* is resident to southern Texas and may be found in other areas of Texas and neighboring states as an occasional summer migrant. In Texas, *H. c. vazquezae* lives in subtropical woodland and scrubland, consuming the passionflowers *Passiflora lutea* L. and *P. affinis* Engelm. (Pyle 1981; Scott 1986; Opler and Malikul 1992). The butterfly is also common in neighboring Mexico where it is found in most vegetation types (Ramirez 1987) and is opportunistic, visiting *Lantana* L. and other plant species common in second growth (Cardoso 2001). *H. c. vazquezae* resides in and around Santa Anna Wildlife Refuge in deep southern Texas, near the Mexican border (SAWR, Hidalgo County [Figure 1]) (Scott 1986; Opler and Malikul 1992). This is the only known location in the US where this subspecies is consistently found (a geographically isolated subspecies, *H. c. tuckeri*, occurs in Florida).

### Arrival reconstruction

Data for the reconstruction of the migration dynamics in Texas were provided by the well known Texas naturalist, Roy Kendall (hereafter RK), who has kept one of the most complete records of butterfly distributions in the state. His notes include information gathered from early Texas travelogues, contributions from fellow naturalists, scientific papers, and his own observations. Because data were collated by one person a possible observer bias may exist. However, with no indication of bias, for the purposes of this study it is assumed that there is none. Also, notes of RK and a more complete and less observer-biased data source (“Butterflies and Moths of North America,” www.butterfliesandmoths.org) agree to a great extent (Figure 1). It is, therefore, assumed that the RK database provides a reasonable picture of *Heliconius charithonia* distribution and a basis for understanding its movement patterns.

Thirty years of presence/absence information are included in the RK data, most of which occurred between 1960 and 1980, but with records dating back to 1884 and as recent as 1992. Some years contain records for only a few areas, while other years contain records for many areas. Thus, sample sizes used in analyses vary depending upon the amount of information provided for any given year. The database comprises a total of 273 observational records from 17 counties in Texas. These notes were converted into a database consisting of locational data (county) and date of observation. If more than one record was available for a location in a given year the earliest and latest records were used to estimate residency time as the interval in months between first and last observation. SAWR was considered the point of entry (source) to calculate the distance from each observation to the source and to examine whether the spatial distribution of butterfly records followed a predictable time-course based on this distance. Distances (km) were measured on a map of Texas (scale: 1:1.472.000).

The data were used to test the following hypotheses: 1) distance from point of entry predicts the date of first butterfly sighting in a county, 2) distance predicts the last date when the butterfly was seen, and 3) distance predicts residency time. Since the city of San Antonio contained the longest time series (18 years [1958–1992], 192 records), the arrival date (measured in Julian days, i.e. the period elapsed from the beginning of the year) was tested to see if it changed during that period.

### Weather and butterfly movements

Because climate has been shown to influence movement patterns, butterfly occurrence was tested (presence/absence in a county) and residency time were predicted by rainfall or temperature. Climate data for rainfall (annual precipitation) is from the Texas Park and the Wildlife Department (www.tpwd.tx.us/publications) and average annual temperatures are from the Southern Regional Climate Center (www.srcc.lsu.edu) for Texas.

### Statistical analysis

Tests of the hypotheses outlined in arrival reconstruction were done with linear regression. To test whether climate influences butterfly occurrence (presence/absence) logistic regression was used. All analyses were performed with JMP 5.0 (SAS Institute 2002).

**Figure 2.**
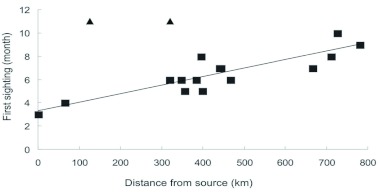
First sighting of *Heliconius charithonia* as a function of distance (km) from presumed source population (Santa Anna Wildlife Refuge, Hidalgo county). The two sites indicated by triangles seem to be outliers (see Results). Line is the regression fit obtained without the outliers. High quality figures are available online.

## Results

### Distance from source and butterfly arrivals

**Figure 3.**
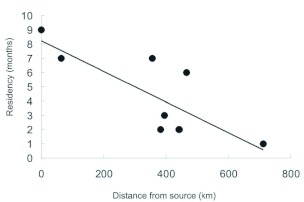
Residency time as a function of distance (km) from presumed source population (Santa Anna Wildlife Refuge, Hidalgo county). Residency times were calculated as the difference between first and last sighting in the county. There is a significant negative linear relationship between residency time and distance from presumed source population. High quality figures are available online.

Butterfly arrivals earlier occur nearer to the source and later further from the source. There was a positive trend, although not statistically significant (Figure 2, r^2^_adj_ = 0.12, F_1,16_ = 3.41, P = 0.08) between distance and sighting. Two outliers (triangles in Figure 2), representing unusual late sightings (late year for Western counties in semi arid region) were removed in order to better explore the data. Removal of the two outliers improved the amount of variance explained by the model (76%) and turned into a highly significant relationship (r^2^_adj_ = 0.76, F_1,14_ = 49.27, P < 0.0001). Distance and last sighting were independent (F_1,16_ = 1.49, P = 0.24). Residency time was inversely related to distance from source (Figure 3, r^2^_adj_ = 0.57, F_1,7_ = 11.8, P = 0.01).

### Long-term trends

First sightings in San Antonio occurred between June and August throughout the interval. There was no discernable trend with regards to changes in first sighting (r^2^_adj_ = 0.05, P = 0.64).

### Climate and butterflies

**Figure 4.**
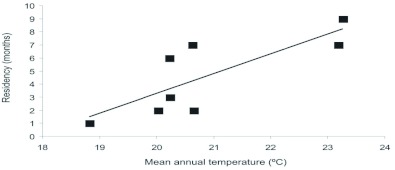
Residency time as a function of mean annual temperature. Residency times were calculated as the difference between first and last sighting in the county. There is a significant positive relationship between time and mean annual temperature. High quality figures are available online.

Butterflies are most often found in warmer locations (19.9±1.9 [SD] °C in occupied vs. 18.0±2.3 °C in unoccupied sites): the logistic regression of temperature and site occupancy was highly significant (χ^2^ = 11.72, df = 1, P = 0.0006). Precipitation, on the other hand, did not predict butterfly presence (738.1±159.8 mm.year^-1^ in occupied vs. 730.4±278.5 mm.year^-1^ in unoccupied sites). Accordingly, the logistic regression did not find a significant model to explain the data (χ^2^ = 0.01, df = 1, P = 0.91). An alternative analysis using rainfall and temperature on a multiple regression yielded the same results, including a non-significant interaction term. Residency length was significantly related to temperature (Figure 4, r^2^_adj_ = 0.57, F_1,6_ = 10.337, P = 0.018).

## Discussion

A tropical butterfly has seasonal movement patterns that are clearly shown by the use of a simple database. From these notes, butterflies are first seen in the south and gradually and predictably they move north. Because data are somewhat fragmented and informal, one might think that they are not useful. For example, in using data from RK political boundaries (county divisions), rather than biology, limit the analysis. Thus, *H. charithonia* is known to stray further than Texas to northern locations such as Oklahoma and Kansas, and better knowledge of arrival dates at these sites could contribute to a better understanding of the migration. However, the inverse relationship between residency time and distance from source suggests that these long distance events may be rare and often go unnoticed. Indeed, even within Texas this species is seldom reported from the northern counties (Figure 1). Thus, despite the informal nature of the data, some important and useful biological information can be found. Indeed, from these data questions arise about the causes and dynamics of these migrations.

What are the mechanisms that drive this northern migration and control its dynamics? Dispersal dynamics in which butterflies are expected to be seen farther from the source as a function of time may cause this pattern. However, if so then a broader distribution pattern is expected with more observations both east and west. This butterfly has been seen at mid-latitudes both east and west, but not in the western arid and semi-arid regions (Figure 1). Also, since northerly sites are consistently “empty” and given the unmistakable color pattern of *Heliconius charithonia* and public interest in butterflies, it is unlikely that these empty areas are due to absence of potential observers. Thus, it is likely that these areas indeed lack the butterfly. This suggests that other factors must influence migration patterns, such as the latitudinal trend in temperature.

If temperature is important, then butterflies may migrate north following the retracting temperature gradient. This may explain the inverse correlation between residency time and distance from source and, consequently, the positive relationship between residency and temperature. No evidence found to date suggests that the butterfly overwinters at these higher latitudes or even that it can withstand colder temperatures. It can be surmised that each year is a new migration and not a new cohort of residents. The northerly expansion of the tropical skipper *Atalopedes campestris* (Boisduval) is constrained by winter temperatures (Crozier 2002, 2003) and it is likely that temperature also limits *H. charithonia*.

Thus, from this simple data source, it can be seen that complex dynamics may drive population movement patterns in this, and other, species. For instance the ecologically similar species, *Dryas iulia* (Fabricius), has a similar distribution to *H. charithonia* distribution in Texas (Figure 1). Each is found in a total of 31 Texan counties. Both species are found in 18 of these counties, an unlikely pattern if their distributions are independent (binomial test, P « 0.01). Given the 254 counties in Texas, one would expect cooccurrence in only three to four counties. This suggests that regardless of the mechanism of *H. charithonia* seasonal dynamics, it is very likely that ecologically similar species have similar dynamics probably due to similar causes.

The analysis for butterflies arriving in San Antonio does not suggest changes over time, although changes in animal and plant phenology due to climate change have been reported from many locations (Roy and Sparks 2000; Forister and Shapiro 2003; Parmesan 2006). The resolution of the data does not allow proper testing of this question. Thus, while this informal dataset is useful in some ways, a more inclusive species database is needed to resolve possible effects of climate change.

## Abbreviatons

RK: Roy Kendall, naturalist
